# Quality of life and service satisfaction in outpatients with severe or non-severe mental illness diagnoses

**DOI:** 10.1007/s11136-018-2039-5

**Published:** 2018-11-03

**Authors:** Marian Ådnanes, Jorid Kalseth, Solveig Osborg Ose, Torleif Ruud, Jorun Rugkåsa, Stephen Puntis

**Affiliations:** 1 Department of Health Research, SINTEF Digital, Klaebuveien 153, 7049 Trondheim, Norway; 2 Division Mental Health Services, Akershus University Hospital, 1478 Lørenskog, Norway; 3 Institute of Clinical Medicine, University of Oslo, Blindern, Box 1171, 0318 Oslo, Norway; 4 Health Services Research Unit, Akershus University Hospital, 1478 Lørenskog, Norway; 5 Centre for Care Research, The University of South-Eastern Norway, 6900 Porsgrunn, Norway; 6 Department of Psychiatry, Warneford Hospital, University of Oxford, Warneford Lane, OX3 7JX Oxford, UK

**Keywords:** Quality of life, Continuity of care, Therapeutic relationship, Unmet service needs, Social relations

## Abstract

**Purpose:**

Our study investigated quality of life (QoL) in patients with severe or non-severe mental illness diagnoses (SMI and non-SMI) and the association between QoL and service satisfaction measured as patients’ perception of continuity of care (CoC), therapeutic relationship, and unmet service needs.

**Methods:**

We conducted a national cross-sectional survey among 3836 mental health outpatients, of whom 1327 (34.6%) responded. We assessed QoL with the Manchester Short Assessment of Quality of Life (MANSA), CoC with the CONTINUUM, the therapeutic relationship with the Therapeutic Relationship in Community Mental Health Care (STAR-P) and developed a simple scale to measure unmet service needs.

**Results:**

Outpatients with SMI (*n* = 155) reported significantly better QoL than those with non-SMI (*n* = 835) (*p* = 0.003). In both groups, QoL was positively associated with cohabitation (*p* = 0.007 for non-SMI and *p* = 0.022 for SMI), good contact with family and friends (*p* < 0.001 for both) and positive ratings of CoC (*p* < 0.001 for non-SMI and *p* = 0.008 for SMI). A positive association between QoL and therapeutic relationship (*p* = 0.001) and a negative association between QoL and unmet needs for treatment (*p* = 0.009) and activity (*p* = 0.005) was only found in the non-SMI group.

**Conclusion:**

Our study highlights the important differences between those with SMI and those with non-SMI in their reported QoL and in the relationship between QoL and service satisfaction, with only non-SMI patients’ QoL influenced by the therapeutic relationship and unmet needs for treatment and activity. It also shows the importance of continuity of care and social factors for good QoL for both groups.

## Background

Modern mental health services not only aim to help patients control their symptoms, but also to help them manage everyday life and to live as good lives as possible [1]. Accordingly, in addition to psychiatric treatment, services often offer assistance across many spheres of patients’ lives, such as housing, employment, receipt of social benefits, meeting places and social activities. Thus, the quality of patients’ lives is seen as a key aim. Continuity of care and good therapeutic relationships with health professionals are seen as fundamental within mental health services and as indicative of the quality of the care provided. However, little is known about whether patients’ quality of life is associated with service satisfaction related to therapeutic relationships, the continuity of the care they receive, or with how well their various service needs are met.

Quality of life (QoL) is increasingly included as an outcome measure in mental health research, often based on Lehman’s definition as “adequate resources, fulfilment of social roles in multiple life domains, satisfaction with life in various domains, and general life satisfaction” [2]. The intention of such a broad perspective is to evaluate a variety of life experiences that can affect the mental health service user’s sense of well-being—areas that, in Lehman’s words, may relate to the need for, and be affected by, the delivery of mental health services [2]. This is a response to deinstitutionalisation of people with mental illness, and the growing need for empirical evidence of QoL among mental health patients living in the community [3]. A subjective QoL approach emphasises the patients’ perspectives and their constant interaction with the environment, and differs from health-related QoL which focuses mainly on health state (physical and psychological) and the consequence of this state for the patient [4]. A recent review of the associations between QoL and service satisfaction in psychotic patients found that subjective QoL yielded stronger associations with service satisfaction than health-related QoL [4].

Patients with mental illness frequently report poorer QoL than the general population [5], and patients in a hospital setting report poorer QoL than patients in community care settings [4]. Differences in subjective QoL between patients with severe mental illnesses (defined here as schizophrenia, schizoaffective disorders and bipolar affective disorders) and non-severe mental diagnoses (such as depression and anxiety disorders)—referred to as SMI and non-SMI, are inconsistent [3]. Several studies show significantly better QoL among patients with SMI diagnoses than among those with non-SMI diagnoses [6–9]. More research is needed both to explore why patients with SMI diagnoses report better QoL than other diagnostic groups and because not all findings can be generalised from one diagnostic group to others [9]. Our study aims to contribute to the latter research question.

Our study’s main focus is on quality of life and service satisfaction measured as the patients’ perception of continuity of care, therapeutic relationship and unmet service needs. These concepts, described more thoroughly in the next paragraphs, and with reference to pertinent literature, reflect highly important components within mental health services: that the patients receive the services that they need, that these services are well coordinated and that the patient has a good relationship with his or her closest provider.

Continuity of care (CoC) is seen as a core value in care delivery [10]. CoC can broadly be described as the long-term delivery of care that is coordinated both within and between services and is appropriate to the patient’s needs [11]. Unique challenges to achieving CoC in mental health services include the need to coordinate treatment across settings and services, the complex health and social needs of patients, and the difficulty of keeping patients who may not want treatment engaged with mental health services. CoC is a multi-dimensional construct that needs to be considered both in the process of care and from the perspective of patients [12]. The present study focuses on patients’ experiences of CoC. Better perceived CoC has been found to be associated with better QoL in a number of studies [6, 7, 11, 13], although these have almost exclusively involved people with SMI as this group is considered more complex and more difficult to achieve continuity with. One exception is Catty and colleagues’ study of people with non-psychotic disorders [6]. They found a greater likelihood of experiencing disruptive and distressing care transitions among persons with non-SMI and concluded that this group’s “relative exclusion from the research agenda should be urgently addressed” (p. 9). Our study aims to further investigate the differences in perceived continuity of care between those with SMI and non-SMI diagnoses.

The relationship between a health professional providing treatment and the person in receipt of that treatment, the feelings and attitudes they have of one another and the way in which they express them is referred to as ‘the therapeutic relationship’. It is considered “the centre of care delivery” [14], and is rated by patients as one of the most important components of mental health care [15]. While a positive therapeutic relationship has been found to be associated with better adherence to treatment and better outcomes [16–18], evidence for the association between CoC and QoL is scarce, especially in relation to those with non-SMI diagnoses. In a review of literature on the therapeutic relationship in the treatment of patients with SMI [19], one study found that positive therapeutic relationship was associated with better QoL among SMI patients who had used services in the long term [20]. Our study aims to explore the association between therapeutic relationship and QoL and investigate the difference in this relationship between patients with SMI and non-SMI diagnoses.

The degree to which a patient’s need for health and social services are met may influence how they evaluate the quality of their lives. Unmet service needs are thus important to consider to improve outcome of interventions regarding quality of life [21]. Patients with reported unmet health and social needs have been found to report poorer QoL [22], and better QoL has been associated and with higher satisfaction with services [4].

Finally, a number of studies establish a link between QoL for persons with mental health problems or illnesses and social support from family and friends [3, 23]. This is highly relevant knowledge in the further development of interventions within mental health services [24].

Our study follows on from a national census of patients receiving outpatient care in Norway, addressing the lack of studies of non-SMI patients in this research area. We had two research questions. First, are there differences in how SMI and non-SMI patients rate their quality of life? Second, are there differences in the association between QoL and service satisfaction in terms of perceived continuity of care, therapeutic relationship and unmet service needs in patients with SMI or non-SMI diagnoses, respectively?

## Methods

### Setting, sample and procedure

In Norway, health care responsibilities are divided between regional health authorities with health trusts that provide specialist services and local authorities (426 municipalities) that deliver primary health and social care. Both levels have developed multi-disciplinary outpatient mental health services over the last 15–20 years. Specialist District Psychiatric Centers (DPC) are located in the community, and municipal mental health teams have been extensively developed based on local circumstances. While both increasingly provide outreach services, this is less common than in other settings such as the UK.

**Fig. 1:**
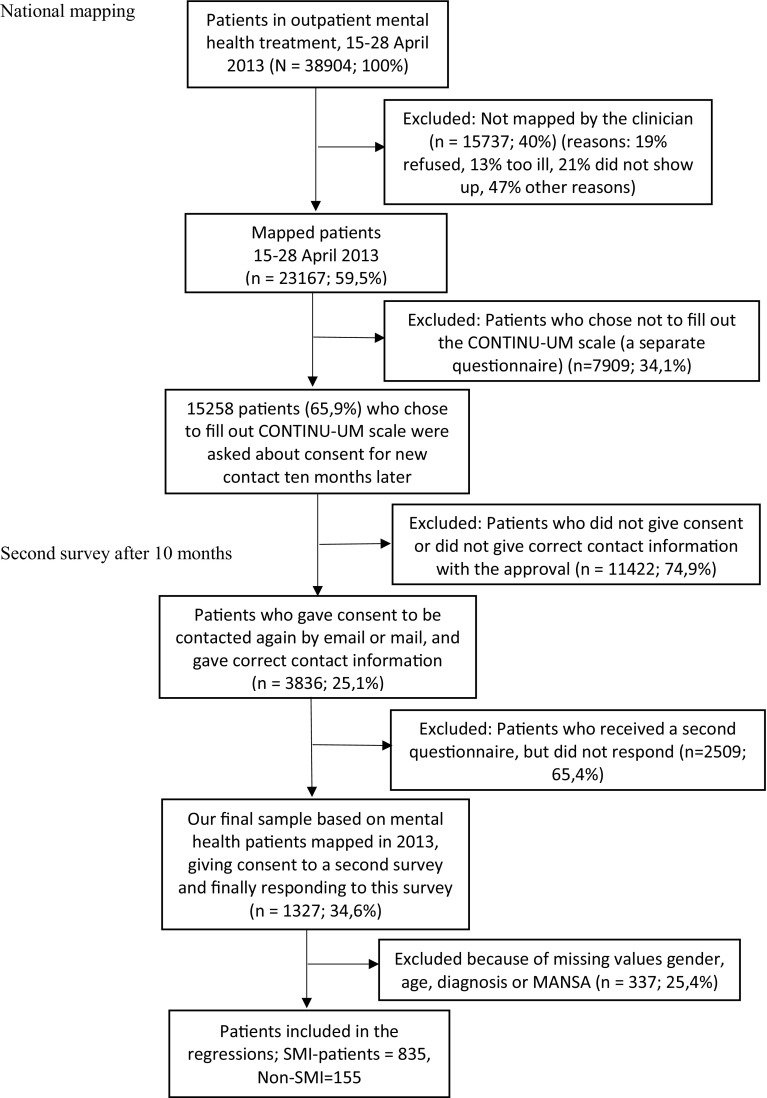
Flow of participants in a national mapping (T1) and a second survey (T2)

On behalf of the Norwegian Directorate of Health, all clinics and departments providing specialist outpatient psychiatric treatment in Norway were approached to participate in the national mapping survey. All outpatients having had at least one treatment contact between 15 and 28 April 2013 were included. In total, 107 of 110 (97,3%) mental health outpatient clinics in Norway participated, and mapping forms were completed and returned for 23,167 outpatients during a 2-week period (see Fig. 1).

Patients who consented to be contacted again were followed up 10 months later with a mailed questionnaire. In this survey, we include all patients who responded to this questionnaire.

### QoL

We used the Manchester Short Assessment of Quality of Life (MANSA) to assess subjective QoL. The MANSA is a brief instrument with a focus on satisfaction with life as a whole and with life domains [25]. According to the authors, the internal consistency of satisfaction ratings was reasonable, and the instrument appears as “viable and valid to obtain condensed and accurate quality of life data” (op cit., p. 10). The authors of the Swedish version of MANSA reported a satisfactory reliability in terms of internal consistency and satisfactory construct validity [26].

We used a translation of MANSA from English to Norwegian, made in agreement with the authors of the scale. We included the individual’s subjective rating of satisfaction concerning 15 of 16 different quality of life domains based on MANSA version 2 and some domains kept from version 1: job, economic situation, social relations, leisure, housing situation, safety, people one lives with, family relations, and physical and psychological health. The question on sexual relations was not included in MANSA version 2 and in our study.

This self-report instrument is rated on a 7-point scale (1, “couldn’t be worse” to 7, “couldn’t be better”). The mean score of domains was used to make the total score. Hence, the range of the total score was from 1 to 7, with higher scores indicating better-rated QoL. Internal consistency was high (Cronbach alpha = .89).

### Socio-demographics

We collected data on gender, age (18–40 years compared to older than 40 years), income (income from employment compared to income from state benefits), education (higher education compared to no education or completed upper secondary school) and living situation (living alone compared to living with others: family or others). Social relations were measured as patient-reported relationships with family and friends (very good and pretty good versus very bad and pretty bad).

The participants were grouped by their diagnosis into either SMI or non-SMI categories. SMI diagnoses in our study included a primary diagnosis of schizophrenia, schizoaffective disorder or bipolar affective disorder (ICD-10 diagnoses F20, F22-F29, F30-F31). Non-SMI diagnoses included the remaining ICD-10 mental illness diagnoses, also referred to as common mental illnesses (for example depression, anxiety disorders, eating disorders and personality disorders). This categorisation of mental illnesses is commonly used in mental health research [27].

### Measures of service satisfaction

#### Continuity of care

We used CONTINUUM to measure patients’ perception of CoC [28]. CONTINUUM has 17 domains (Table 4), each comprising three items: (i) the importance of the domain; (ii) ease of access to the domain if needed; (iii) how satisfied the respondent was with their experience. The authors report satisfactory psychometric properties [28]. For our purpose we only included (ii) ease of access to the domain. In agreement with the authors of the scale, we translated it from English to Norwegian.

Patients’ satisfaction with each domain is scored on a five-point Likert scale (1–5). We added a sixth category in our Norwegian version. The patient could choose “not relevant/ no need” if access to the domain was irrelevant to him/her.

We included 13 of the 17 domains in our analysis. Three were removed because the majority of participants had replied “not relevant/no need”. These were questions about previous hospital discharge (irrelevant for 65.6% of participants), attending day centres (irrelevant for 62.8% of participants) and having a care plan (irrelevant for 50.2% of participants). A fourth domain was removed because it was considered difficult to interpret (“able to avoid services”). The mean score of domains was used to make the total score. Hence, the range of the total mean score was from 1 to 5, with higher scores indicating better-rated CoC. Internal consistency was good (Cronbach’s alpha = .87).

#### Therapeutic relationship

The Scale to Assess the Therapeutic Relationship in Community Mental Health Care—service user version (STAR-P) [14], was used to assess the relationship between the service user and their “closest practitioner”, that is, the person in the treatment team that the patient rates as most important to them. STAR has good psychometric properties and the authors have reported good test–retest reliability [14]. The psychometric properties in a German version of STAR-P has been tested and found acceptable [29].

We used a translation of STAR-P from English to Norwegian, made in agreement with the Nordic co-author of the scale. STAR-P has 12 items comprising three subscales: positive collaboration, positive clinician input and non-supportive clinician input. Each item is scored on a five-point scale (0–4) with higher scores indicating a better therapeutic relationship. Hence, the total mean score was 0–4. Internal consistency was good (Cronbach’s alpha = .89).

#### Unmet need for services

We assessed patient-rated unmet service needs through a questionnaire developed for the national mapping exercise. It required patients to consider 24 different services and indicate (a) their need for that service (yes/no) and (b) whether they received that service (yes/no). Two new dichotomous variables were constructed, respectively, based on items for treatment and activity. An unmet need for treatment was coded either one (for ‘yes’) if the patient had an unmet need for treatment in either a specialist outpatient service or with municipal mental health services, or zero (for ‘no’) if they did not have an unmet need. An unmet need for activity was coded one if the patient had unmet need in either work measures, activity center/day center, group support or individual support contact, meeting places or user-driven selfhelp groups. Unmet need for each of these services ranged from 3.2% to 9.8% of the participants.

### Statistical analyses

We calculated descriptive statistics for all variables to obtain and compare means and standard errors for the SMI and non-SMI groups and conducted *t* tests to test differences between the groups (Table 1). To assess the representativity of the study population, the two groups’ profiles at Time 1 and Time 2 were calculated, and a test of differences between the two time points was performed (Table 5). Linear bivariate (Table 2) and multivariate regressions (Table 3) for SMI and non-SMI were performed to assess the association between QoL, measured by MANSA, on the following groups of variables: socio-economic background, social relations, continuity of care, therapeutic relation and unmet service needs. *p* values below 0.05 were considered statistically significant. Stata version 14.0 for Windows was used for the analysis.

**Table 1:** Demographic characteristics, quality of life (QoL), continuity of care (CoC), therapeutic relationship and unmet service needs (%, n), and test of differences between participants with non-severe mental illnesses (non-SMI; *n* = 835) and severe mental illnesses (SMI; *n* = 155) (*χ*^2^)

	Non-SMI (*n* = 835)	SMI (*n* = 155)	*χ* ^2^ Non-SMI versus SMI *p* =
	% (*n*)	% (*n*)	
Gender (1 = women)	76.3 (637)	63.9 (99)	0.001
Age (1 = 40+)	39.9 (333)	52.9 (82)	0.003
Education (1 = above secondary school)	50.4 (421)	54.8 (85)	0.312
Income (1 = own income from labour)	36.8 (307)	23.9 (37)	0.002
Living situation (1 = do not live alone)	72.2 (603)	59.4 (92)	0.001
Contact with family (1 = good)	77.5 (647)	87.1 (135)	0.007
Contact with friends (1 = good)	77.0 (643)	80.0 (124)	0.413
Unmet need for treatment (1 = unmet)	12.2 (102)	10.3 (16)	0.504
Unmet need for activity (1 = unmet)	18.1 (151)	19.4 (30)	0.707

	Mean (SE)	Mean (SE)	*t* test Non-SMI versus SMI *p* =
QoL (MANSA, score 1–7)	4.329 (0.038)	4.606 (0.081)	0.003
Therapeutic relationship (STAR, score 0–4)	3.046 (0.024)	3.065 (0.053)	0.747
Continuity of care (CONTINUUM, score 1–5)	3.309 (0.031)	3.400 (0.068)	0.244

**Table 2:** Linear bivariate regressions for association between quality of life (QoL) and continuity of care (CoC), therapeutic relationship, unmet service needs and background variables for participants with non-severe mental illnesses (non-SMI; *n* = 835) and severe mental illnesses (SMI; *n* = 155)

Variable	Non-SMI			SMI		
	Coef.	*p* > |*t*|	[95% CI]	Coef.	*p* > |*t*|	[95% CI]
Gender (1 = women)	0.167	0.060	− 0.007 to 0.167	0.195	0.252	− 0.140 to 0.529
Age (1 = 40+)	0.093	0.229	− 0.059 to 0.093	0.064	0.696	− 0.259 to 0.387
Education (1 = above secondary school)	0.449	0.000	0.300–0.449	− 0.211	0.211	− 0.544 to 0.121
Income (1 = own income from labour)	0.338	0.000	0.184–0.338	0.232	0.225	− 0.144 to 0.608
Living situation (1 = do not live alone)	0.277	0.001	0.112–0.277	0.300	0.070	− 0.025 to 0.624
Contact with family (1 = good)	0.909	0.000	0.741–0.909	1.551	0.000	1.132–1.969
Contact with friends (1 = good)	1.280	0.000	1.125–1.280	1.241	0.000	0.882–1.600
Therapeutic relationship (STAR)	0.639	0.000	0.539–0.639	0.611	0.000	0.385–0.837
Continuity of care (CONTINUUM)	0.586	0.000	0.514–0.586	0.557	0.000	0.389–0.725
Unmet need for treatment (1 = unmet)	− 0.860	0.000	− 1.079 to − 0.860	− 0.528	0.048	− 1.051 to − 0.004
Unmet need for activity (1 = unmet)	− 0.754	0.000	− 0.940 to − 0.754	− 0.603	0.003	− 1.000 to − 0.207

**Table 3:** Linear multivariate regression for association between quality of life (QoL) and continuity of care (CoC), therapeutic relationship, unmet service needs and background variables for participants with non-severe mental illnesses (non-SMI; *n* = 835) and severe mental illnesses (SMI patients; *n* = 155)

Variable	Non-SMI			SMI		
	Coef.	*p* > |*t*|	[95% CI]	Coef.	*p* > |*t*|	[95% CI]
Gender (1 = women)	0.036	0.600	− 0.099 to 0.171	0.135	0.286	− 0.115 to 0.385
Age (1 = 40+)	0.000	1.000	− 0.116 to 0.116	0.166	0.185	− 0.081 to 0.413
Education (1 = above secondary school)	0.348	0.000	0.232–0.464	− 0.049	0.716	− 0.314 to 0.216
Income (1 = own income from labour)	0.082	0.177	− 0.037 to 0.202	− 0.122	0.412	− 0.415 to 0.171
Living situation (1 = do not live alone)	0.174	0.007	0.048–0.299	0.285	0.022	0.041–0.528
Contact with family (1 = good)	0.411	0.000	0.269–0.553	1.119	0.000	0.737–1.502
Contact with friends (1 = good)	0.839	0.000	0.695–0.982	0.750	0.000	0.425–1.075
Therapeutic relationship (STAR)	0.183	0.001	0.076–0.291	0.147	0.229	− 0.094 to 0.389
Continuity of care (CONTINUUM)	0.280	0.000	0.191–0.369	0.268	0.008	0.070–0.466
Unmet need for treatment (1 = unmet)	− 0.242	0.009	− 0.425 to − 0.060	0.017	0.937	− 0.407 to 0.441
Unmet need for activity (1 = unmet)	− 0.226	0.005	− 0.382 to − 0.069	− 0.184	0.290	− 0.525 to 0.158
Constant	1.594	0.000	1.277–1.910	1.414	0.000	0.698–2.129

### Missing values

Our data contained missing observations on most variables. For the background variables (age, gender, education, income, living situation, diagnosis, contact/relation with family and friends), the proportion of missing observations varied from 3.2% for gender and family to 7.5% for diagnosis. Both the outcome measure (MANSA) and two covariates (STAR and CONTINUUM) are multi-item scales with missing data on one or more items. For items in MANSA and STAR, the proportion of missing was 8.5–9.3% and 12.7%–14.1%, respectively. The problem of missing data was considerable for some items in the CONTINUUM scale, varying from 15.7% for item 2 to 57.1% for item 5 (items 6, 11, 12 and 16 were excluded). For MANSA and STAR, most of the missing observations were due to missing data on all items (107 for MANSA, 164 for STAR, 164 for CONTINUUM).

Missing data were estimated using multiple imputation by chained equations (with 100 imputations), applying the MI procedure in STATA (version 14.0) and pooled in the final regression model using Rubin’s rule [30]. All variables in the final regression model were included in the imputation equations. However, the imputations failed to converge when all items were included in all equations. We therefore used a variant of the approach suggested by Plumpton et al. [31] which reduces the number of variables in the imputation model while imputing the individual items. We excluded cases with missing values on age, gender and diagnosis group. Age, gender and diagnosis group were retained in the imputation model as regular variables as were the unmet needs variables. Individuals with missing MANSA items were excluded in the final regression [32].

## Results

### Participants

Of the 23,167 patients participating in the national mapping exercise, 3836 (25.1%) agreed to be contacted again after some months via email or mail with a new questionnaire. 1327 (34.6%) of these patients responded to the second questionnaire. 337 participants were excluded because of missing data on gender, age, diagnosis or MANSA. 990 of 1327 (74.6%) participants were included in the regressions, 155 (15.7%) with SMI and 835 (84.3%) with non-SMI diagnosis.

There were significant differences (*p* < 0.05) between the SMI sample and the non-SMI sample (Table 1). In both samples the majority were female, but in the non-SMI sample the proportion of women were significantly higher than in the SMI sample (*p* = 0.001). In the SMI sample, more participants were above 40 years old than in the non-SMI sample (*p* = 0.003). There were no significant differences in education, but in the non-SMI group, more had income from work compared to the SMI group (*p* = 0.002). More people in the non-SMI group lived together with someone than in the SMI group (*p* = 0.001). In the SMI group, significantly more people reported good contact with their family than in the non-SMI group (*p* = 0.007). There were no significant differences in contact with friends.

There were significant differences between sample characteristics both in the SMI and the non-SMI sample at T2 compared to T1 (Table 5). More women than men participated in T2 compared to T1. In the non-SMI sample, participation of young people in the age groups 18–29 years dropped significantly, the share of participants without any education (secondary or higher) decreased, and more persons with their own income participated in the second survey. The share of participants living alone decreased significantly from T1 to T2 in the SMI sample, while in both samples the share of participants living with spouse or cohabitant increased. In the SMI sample, the share of participants with good contact with friends increased significantly from T1 to T2.

### The association between QoL and socio-demographics in patients with a diagnosis of SMI or non-SMI

The mean QoL (MANSA) score for patients with an SMI diagnosis was significantly higher than those with a non-SMI diagnosis (*p* = 0.003) (Table 1).

Linear bivariate regressions for non-SMI and SMI patients are presented in Table 2, and the multivariate regression results are presented in Table 3. The bivariate results show a significant association between QoL and service satisfaction, measured as therapeutic relationship, CoC and unmet service needs for both non-SMI and SMI patients. In the multivariate regressions, only CoC remains significant in the SMI group, due to a combination of correlated service satisfaction measures and the small sample of patients with SMI. CoC and STAR are positively correlated, while both are negatively correlated with unmet needs.

QoL was not significantly associated with sociodemographic variables among outpatients with SMI diagnosis (Table 3). Non-SMI patients with higher education reported significantly better QoL than those with only a secondary school or lower education (*p* < 0.001). Furthermore, persons living together with someone reported significantly better QoL compared to persons living alone. This was found for both non-SMI (*p* = 0.007) and SMI (*p* = 0.022) groups. Having good contact with family and friends was also significantly associated with better QoL for both patient groups (*p* < 0.001 for both).

### The association between QoL and service satisfaction measured as CoC, therapeutic relationship and unmet service needs

Among non-SMI patients, QoL was positively associated both with the patient’s perception of good therapeutic relationship with their closest service provider (*p* = 0.001) and with their perception of CoC (*p* < 0.001) (Table 3). SMI patients’ QoL were positively associated with CoC (*p* = 0.008), but not with therapeutic relationship. Furthermore, in non-SMI, QoL was negatively associated with unmet needs for treatment (*p* = 0.009) and activity (*p* = 0.005).

## Discussion

The aim of the study was to investigate quality of life among outpatients with severe or non-severe mental illnesses, and the association between QoL and service satisfaction measured as perceptions of continuity of care, therapeutic relationship and unmet service needs. The results show better QoL among patients with SMI diagnoses. In both patient groups, QoL was positively associated with good social relations and continuity of care. In the non-SMI group QoL was also positively associated with good therapeutic relationship and negatively associated with unmet needs for treatment and activity.

### QoL in SMI and non-SMI patients and association with socio-economics and social relationship

Those with a SMI reported better QoL than those with non-SMI in our study, which is a regularly reported finding [6–9]. One explanation for this difference is that patients with schizophrenia adapt differently than patients with other disorders, either because they have lowered expectations leading to higher satisfaction or because of their clinical characteristics [9]. Emotional withdrawal, affective blunting or cognitive deficits may limit the impact of higher symptom levels on QoL [33]. For those with non-SMI, poorer perceived QoL could also be a proxy for mood [6].

We did not find an association between QoL and employment in patients with SMI, although there was a non-significant trend in the non-SMI group. Employment status may not influence QoL in patients with schizophrenia as much as it does in those with mood disorders and neurotic disorders due to lack of fulfilling work in those with SMI [9]. Educational status on the other hand was significantly positively associated with QoL in the non-SMI group.

Social relationships are considered a strong predictor for good QoL [3], and associations have been found between the characteristics of social networks, such as satisfaction with social contacts [24, 34] and the availability of close personal relationships that permit emotional integration [35]. However, the importance of relatives and friends found in other studies mostly focus on patients with schizophrenia living in the community [24]. Our study confirms the importance of social networks for both patients with and without SMI. We found an association between QoL and living together with a husband or wife, as well as for contact with family and friends. We also found differences between the groups for the relative importance of family versus friends. For SMI patients, good contact with family was more strongly associated with QoL than good contact with friends. The opposite was true for non-SMI, with friends being more important than family. This may be due to the isolating nature of SMI, indicated by smaller social networks, and a smaller number of friends compared to family in their network [36]; thus, the reliance on family for social support might be much greater.

There is now a body of evidence which points to a need to focus on interventions concerning patient’s social relationships through strengthening of social support, the use of family and friends, consideration of informal caregivers’ needs, and a focus on interventions which enhance the patient’s social world [24, 34, 35, 37].

### QoL’s association with service satisfaction measured as CoC, therapeutic relationship and unmet service needs

Our study found that those with non-SMI diagnoses reported poorer QoL than those with SMI diagnoses, but did not report a significantly lower rate of patient-rated continuity of care (CoC) and therapeutic relationship. The groups differed in the extent of the association between service satisfaction and their reported QoL. Among non-SMI patients, QoL was significantly associated both with perceived CoC in services, the therapeutic relationship and unmet service needs. Among SMI patients, QoL was only associated with CoC. Catty and colleagues found a significant association between CoC and change in clinical or social outcomes in their psychotic cohort, but not in the non-psychotic cohort [6, 7]. However, their study investigated changes in QoL’s association with changes in CoC and therapeutic relationship over 12 months, while ours only compared these at a single time point.

Unmet service needs are not reported to a large extent among the participants in our study. Still, unmet needs for mental health treatment and activities were significantly associated with poorer QoL among those with a non-SMI diagnosis, which has previously been identified in both UK and the Nordic samples [22, 34, 38, 39].

Our study found interesting differences between the two patient groups. There was a strong relationship between service satisfaction and perceived subjective QoL in the non-SMI group. Patients with non-SMI diagnoses may be more closely connected to their providers, for example through better communication, than patients with SMI diagnoses. Non-SMI patients had a higher socioeconomic status than the SMI group and this may influence the communication between patient and provider [40].

We also found that having access to more everyday activities was associated with a better quality of life. A review has previously found that meeting unmet service needs and strengthening the social support of the clients influence QoL [41]. Slade and colleagues have also argued that meeting patient-rated unmet needs should be the starting point for mental health care [22], and involving patients’ subjective perspectives of their QoL in the treatment process positively influences patient satisfaction, confirming the relevance of measuring QoL in clinical practice [42].

### Strengths and limitations

The major strengths of our study are a fairly large sample, collected across the whole country, and covering a broad range of outpatients. Standardised instruments with good psychometric properties have been used. The instruments are, however, not standardised with the establishment of standard values for different populations, but users with mental disorders have helped develop them, and the instruments have been used in a variety of studies and with several patient groups [6, 7, 9].

There are limitations. Our sample of non-SMI patients is much larger than the sample of SMI patients, although it is broadly representative of the prevalence of mental illness in the population. It is an exploratory and cross-sectional study, and we do not conclude causality between variables that are associated with each other.

Our study contained missing data for most variables, with much missing data for items in the CONTINUUM scale. We have tried to address this by using multiple imputation.

Even though our study includes key factors for service satisfaction within mental health discussed in the literature, there may be omitted-variable bias as a result of unmeasured service characteristics that both impact QoL and are correlated with the included variables.

Females are also over-represented in our sample and participants in T2 were better educated, more were employed, and there were fewer persons living alone and without social networks, which may represent some degree of participation bias.

Finally, QoL has been critiqued due to a lack of a consensus definition, a lack of a ‘gold-standard’ measurement and the potential for subjective QoL measurement distortion due to altered mood states. However, despite these weaknesses there is evidence that subjective QoL can be used as valid as measure in psychiatric research without being compromised by the patient’s illness [43].

### Conclusion

This study highlights important differences between SMI and non-SMI patients in their reported QoL and in the association between QoL and service satisfaction, with only non-SMI patients’ QoL influenced by the therapeutic relationship and having unmet needs for treatment and activity. For both patient groups, the study shows the importance of continuity of care and social factors for good QoL.

The findings indicate that patients with SMI have a weaker connection with the health services. There is a need to aim for more involvement of this patient group and their subjective perspectives in the treatment process as research show that this positively influences patient satisfaction and improves their quality of life. For both groups, the findings related to the social factors in our study points to the importance of relatively simple actions of everyday life, for example strengthening of social support and more targeted use of family and friends, and to focus on interventions which enhance the patient’s social world.

## Appendix

See Tables 4 and 5.

**Table 4:** Names and descriptions of the 17 domains in CONTINUUM

Domain name	Domain description
Accessing services	Been able to easily access services when you’ve needed to
Range of services	Been able to get all the services you feel you need
Choice	Had choice over the types of treatments you’ve received
Waiting	Had to wait a long time to receive services
Out of hours support	Have you had access to support from services outside of office hours
Hospital discharge	Received the support you’ve needed from services when you have left hospital
Staff changes	Have the staff involved in your care changed frequently
Information	Been able to get appropriate information from staff
Flexible levels of support	Have the levels of support you get from services changed to match your needs
Individual progress	Have the services you’ve received helped you to move forward
Day centres	Had access to day centres that suit your needs
Care plans	Agreed with your care plan
Crisis	Had systems in place for dealing with a crisis
Communication between staff	Have the staff involved in your care seemed to communicate with each other
Support from other users	Had support from other people who have experienced mental distress
Repeating your life history	Had to tell your life history to new staff
Avoiding services	Been able to avoid contact with services if you have wanted to

**Table 5:** Participants’ characteristics (percent, number in parentheses) among participants with non-severe mental illnesses (non-SMI) and severe mental illnesses (SMI) at Time 1 (*n* = 15,258) and Time 2 (*n* = 1327), and Likelihood ratio (LR) Chi-square test between Time 1 and Time 2 in the two groups

Category	Time 1 only Percent (*n* = 13,033^a^)		Time 1 and Time 2 Percent (*n* = 1227^a^)		Test Likelihood ratio (LR) Chi-square test Time 1 only and Time 1 and Time 2	
	Non-SMI	SMI	Non-SMI	SMI	Non-SMI	SMI
Total	83.1 (10,832)	16.9 (2201)	84.3 (1035)	15.7 (192)	LR *χ*^2^(1) = 1.26 *p* = 0.2625	
Sex						
Male	32.7 (3506)	49.0 (1068)	25.6 (262)	36.7 (69)	LR *χ*^2^(1) = 22.39 *p* < 0.000	LR *χ*^2^(1) = 10.61 *p* = 0.001
Female	67.3 (7213)	51.0 (1112)	74.4 (761)	63.3 (119)		
Age (years)						
18–23	17.4 (1828)	8.3 (177)	11.6 (115)	7.0 (13)	LR *χ*^2^(6) = 61.58 *p* < 0.000	LR *χ*^2^(6) = 8.56 *p* = 0.198
24–29	18.8 (1972)	16.1 (343)	18.0 (179)	16.2 (30)		
30–39	25.5 (2672)	28.4 (606)	30.3 (301)	24.3 (45)		
40–49	20.8 (2185)	23.4 (499)	25.4 (252)	27.6 (51)		
50–59	10.9 (1142)	15.8 (336)	11.6 (115)	14.6 (27)		
60 and older	6.5 (684)	8.0 (170)	3.1 (31)	10.3 (19)		
Education						
Not completed upper sec. school	27.0 (2817)	30.5 (642)	16.3 (163)	12.0 (22)	LR *χ*^2^(2) = 117.2 *p* < 0.000	LR *χ*^2^(2) = 37.84 *p* < 0.000
Completed upper sec. school	39.4 (4110)	36.1 (761)	33.7 (338)	36.8 (67)		
Started or completed university degree	33.6 (3500)	33.4 (705)	50.0 (501)	51.1 (93)		
Income						
Own income from labour	33.6 (3539)	16.9 (364)	36.6 (369)	25.5 (48)	LR *χ*^2^(1) = 3.75 *p* = 0.053	LR *χ*^2^(1) = 8.08 *p* = 0.004
Not own income from labour	66.4 (7003)	83.1 (1788)	63.4 (639)	74.5 (140)		
Living situation						
Living alone	27.7 (2970)	50.4 (1094)	28.4 (290)	42.3 (79)	LR *χ*^2^(5) = 19.36 *p* = 0.002	LR *χ*^2^(5) = 14.91 *p* = 0.011
Living alone with children	11.1 (1185)	6.5 (141)	10.3 (105)	7.5 (14)		
Living with parents	12.1 (1300)	9.7 (211)	8.0 (82)	7.0 (13)		
Living with married/cohabitant	19.4 (2077)	14.2 (307)	20.5 (210)	20.9 (39)		
Living with married/cohab/child	24.6 (2639)	14.1 (305)	27.4 (280)	19.8 (37)		
Living with other	5.1 (541)	5.1 (111)	5.5 (56)	2.7 (5)		
Social network						
Good contact with family	82.7 (8638)	85.7 (1821)	82.4 (816)	83.3 (155)	LR *χ*^2^(1) = 0.05 *p* = 0.817	LR *χ*^2^(1) = 0.72 *p* = 0.397
Not good contact with friends	17.3 (1805)	14.3 (305)	17.6 (174)	16.7 (31)		
Good contact with friends	80.7 (8395)	74.0 (1548)	82.3 (813)	83.3 (155)	LR *χ*^2^(1) = 1.46 *p* = 0.228	LR *χ*^2^(1) = 8.57 *p* = 0.003
Not good contact with friends	19.3 (2005)	26.0 (544)	17.7 (175)	16.7 (31)		

^a^The number of participants on Time 1 was reduced from 15,258 to 13,033, and on Time 2 from 1337 to 1227 due to missing values on the diagnosis variable
